# Satellite Tracking Reveals Long Distance Coastal Travel and Homing by Translocated Estuarine Crocodiles, *Crocodylus porosus*

**DOI:** 10.1371/journal.pone.0000949

**Published:** 2007-09-26

**Authors:** Mark A. Read, Gordon C. Grigg, Steve R. Irwin, Danielle Shanahan, Craig E. Franklin

**Affiliations:** 1 Queensland Parks and Wildlife Service, Cairns, Queensland, Australia; 2 School of Integrative Biology, The University of Queensland, St. Lucia, Queensland, Australia; 3 Australia Zoo, Beerwah, Queensland, Australia; Dalhousie University, Canada

## Abstract

Crocodilians have a wide distribution, often in remote areas, are cryptic, secretive and are easily disturbed by human presence. Their capacity for large scale movements is poorly known. Here, we report the first study of post-release movement patterns in translocated adult crocodiles, and the first application of satellite telemetry to a crocodilian. Three large male *Crocodylus porosus* (3.1–4.5 m) were captured in northern Australia and translocated by helicopter for 56, 99 and 411 km of coastline, the last across Cape York Peninsula from the west coast to the east coast. All crocodiles spent time around their release site before returning rapidly and apparently purposefully to their capture locations. The animal that circumnavigated Cape York Peninsula to return to its capture site, travelled more than 400 km in 20 days, which is the longest homeward travel yet reported for a crocodilian. Such impressive homing ability is significant because translocation has sometimes been used to manage potentially dangerous *C. porosus* close to human settlement. It is clear that large male estuarine crocodiles can exhibit strong site fidelity, have remarkable navigational skills, and may move long distances following a coastline. These long journeys included impressive daily movements of 10–30 km, often consecutively.

## Introduction

*Crocodylus porosus* is the world's largest crocodilian and has the widest geographical range of any, occurring from the Solomon Islands to Papua New Guinea and across northern Australia to Indonesia, south-east Asia and the eastern coast of India [1]. It is also the most dangerous [2]. Managing problem *C. porosus* by translocation to a remote location is of dubious value because many of them return [3], but translocation is still suggested in the public domain as a way to ameliorate the threat posed by large crocodiles in areas close to human habitation. Data on homing by translocated crocodilians has been gathered mostly by surveillance at the original capture site for the return of tagged individuals [3]–[7]. These studies indicate that crocodilians are likely to show site fidelity. However, the drawback of mark-translocate-recapture studies is that they provide no information about the tracks taken by returning individuals, or the time profile of the journey. For this, telemetry is necessary.

There have been several attempts at following translocated crocodilians by radiotelemetry, over short distances and short time frames [7]. Rodda (1984) reported radiotelemetry data from hatchling and juvenile *Alligator missippiensis* that implied a capacity for navigation because they homed after translocation short distances (1–7 km) to an unfamiliar area [8]. However, studying movement patterns in crocodilians by conventional radiotelemetry has proven difficult because they are mostly very cryptic, live in remote locations, have wide geographic ranges and are easily disturbed by human presence. Satellite tracking, on the other hand, allows data to be collected essentially continuously from animals in remote locations that are difficult to access, such as along the coastline and in the open ocean, and without the human interference which is hard to avoid during manual tracking. Satellite telemetry has been very successful for studying movements of various birds, mammals, fish, and marine turtles [9]–[12]. Surprisingly, there have so far been no published studies in which satellite telemetry has been used to study the movements of crocodilians. In reporting the results of his study of the movement of *C. porosus* in the Cambridge Gulf, northern Australia, Kay (2004) referred to the limitations of conventional VHF telemetry and recommended the use of satellite tracking in future studies in order to gather data over larger spatial and temporal scales.

The aims of our study were to record and interpret the movements of translocated large male estuarine crocodiles after their release and to investigate their homing behaviour, if any, using satellite telemetry.

## Methods

**Figure 1:**
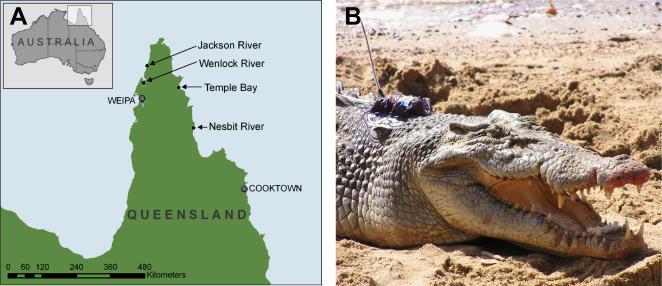
Location of study sites and position of a satellite transmitter on an estuarine crocodile. A Map of Queensland, Australia showing the study sites and capture locations of the three crocodiles that were translocated. B A large, male estuarine crocodile (*Crocodylus porosus*) with a satellite transmitter attached between the nuchal scutes.

Three large male estuarine crocodiles, *Crocodylus porosus* (3.1–4.5 m) were captured on the Nesbit and Wenlock Rivers, Cape York Peninsula, North Queensland, Australia (Table 1, Fig. 1A). These sites were chosen because they contain healthy populations of large estuarine crocodiles [13]. Crocodiles were captured using standard trapping methods [14] and restrained while they were measured and had satellite transmitters fitted.

**Table 1:** Summary of data for translocated, male estuarine crocodiles, *Crocodylus porosus*

Croc	Length (m)	River	Translocation Distance (km)	Date of Translocation	Minimum distance to travel to return to capture location (km)	Date Arrived Home
A	3.8	Wenlock	77	25/8/2004	99	20/9/2004
B	3.1	Nesbit	52	14/9/2003	56	5/10/2003
C	4.5	Wenlock	126	16/8/2004	411	24/12/2004

The satellite transmitters were KiwiSat101 platform terminal transmitters (PTT) (Sirtrack; Lower Hutt, New Zealand) powered by a C-sized lithium battery with a duty cycle of 24 h on, 72 h off and a repetition rate of 60 s. The electronics were packaged into epoxy resin with the flexible antenna for the PTT exiting the top surface of the package at 45 degrees posteriorly. The overall dimensions for each PTT were approximately 120 mm (L)×32 mm (W)×24 mm (H) and a mass of 300 g.

Satellite transmitters were attached between the nuchal scutes following a protocol similar to that described by Kay (2004). The transmitters were attached with plastic-coated braided stainless steel wire threaded through small holes drilled horizontally through the osteoderms of the nuchal shield. The wire was threaded through two loops on each side of the transmitter, drawn up to hold the transmitter closely in place but not tightened, and secured with crimps (Fig. 1B). Prior to drilling, a local anaesthetic (Lignocaine) was infiltrated under the nuchal shield to numb the area. Shortly after transmitters were fitted, the crocodiles were moved from their capture locations to new locations (Table 1) by suspending them under a helicopter in a net sling. Crocodiles were translocated to appropriate sites in which other crocodiles were present. We could not determine the density of crocodiles in the surrounding area but it is generally regarded that the population densities of large *C. porosus* on Cape York peninsula is low [13] due to past hunting practices.

The locations of the crocodiles after release were recorded by the Argos satellite system. Positions with Argos accuracy Classes 1, 2 or 3 (Table 2) were used within this study, as this provided data with suggested accuracy of less than one kilometre (Argos User's Manual, 2000) [15]. Data was mapped in ArcGIS 9 against river and coastline vector layers to confirm general accuracy of data. Distances moved between consecutive location records were calculated using the Hawth's Tools extension to ArcGIS 9 [16]. The per-day distance moved was calculated by dividing the total distance between consecutive points by number of days passed (hence average distances moved are calculated for 24 hour periods).

**Table 2:** Argos location classes and number of fixes for three male *Crocodylus porosus*

Argos Location Class	Estimated Accuracy	Number of positions		
		Crocodile A (25/8/04-25/11/04)	Crocodile B (14/9/03-31/11/03)	Crocodile C (16/8/04-23/1/04)
3	<150 m	14	46	18
2	150 m to 350 m	26	41	18
1	350 m to 1000 m	22	30	26
0	>1000 m	12	8	36
A	No estimate of accuracy	12	25	25
B	No estimate of accuracy	26	28	25
Z	Invalid locations	44	41	40

Location classes 0, A, B & Z were excluded from our data analysis.

## Results

**Figure 2:**
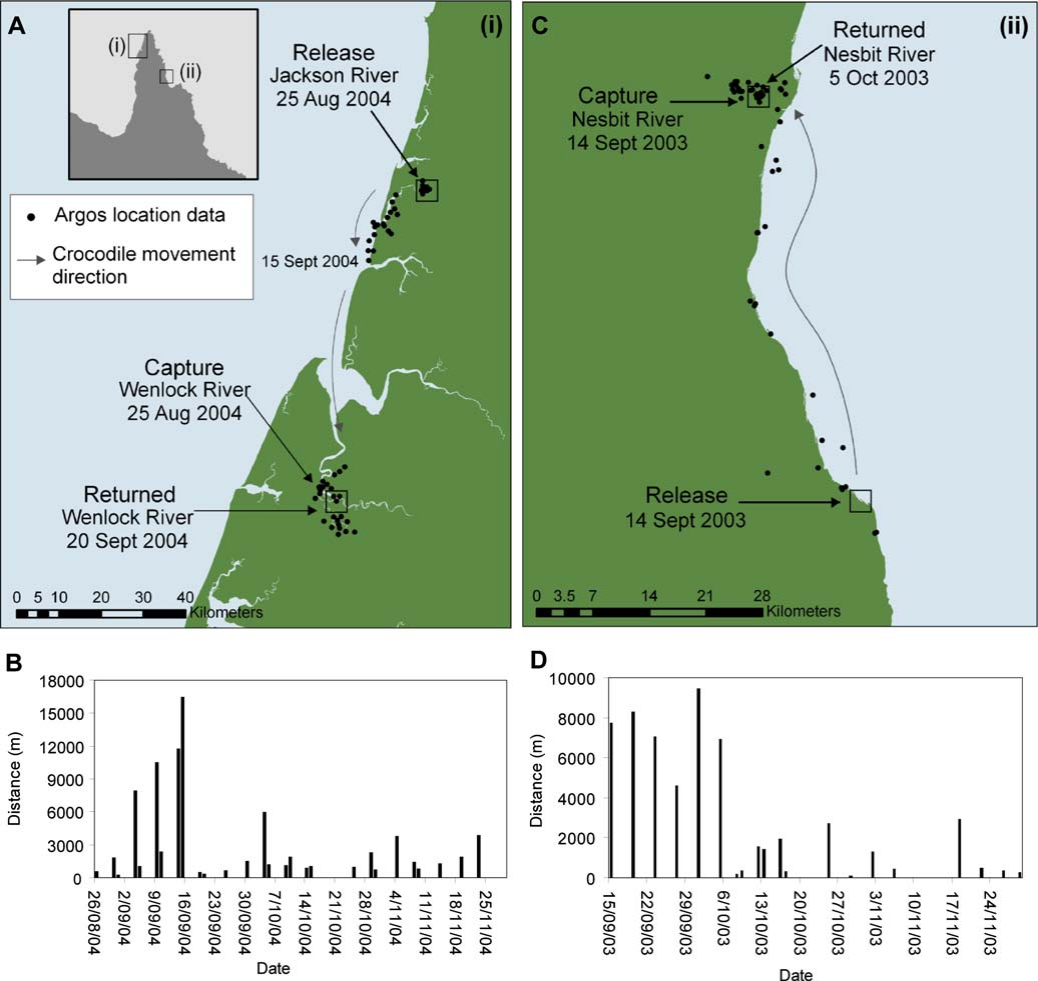
Movement patterns of two translocated estuarine crocodiles. A Map and location fixes of Crocodile A captured in the Wenlock River and flown 77 km north to the Jackson River. B Daily distances covered (m) by Crocodile A after release. C Map and location fixes of Crocodile B captured in the Nesbit River and flown 52 km south and released into the ocean. D Daily distances covered (m) by Crocodile B after release.

**Figure 3:**
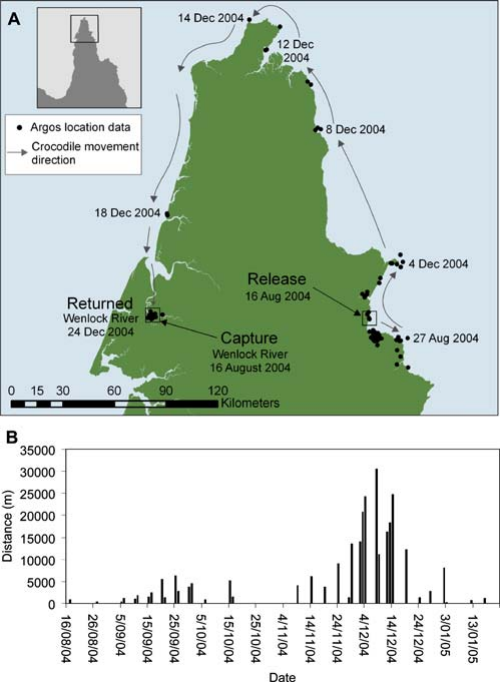
Movement patterns of the translocated estuarine crocodile that circumnavigated Cape York Peninsula. A Map and location fixes of Crocodile C captured in the Wenlock River on the west coast and flown across Cape York Peninsula and released into the ocean on east coast. B Daily distances covered (m) by Crocodile C after release.

All three crocodiles returned to their original capture sites (Fig. 2 & 3). They all behaved similarly after release, each making apparently random movements around the release site for periods between 10 and 108 days, and then taking the most direct coastal route back to their capture sites. Once back at their capture sites, all showed strong site fidelity, remaining in that vicinity for the remainder of the tracking period.

In more detail, Crocodile A was captured on 25 August 2004 in the Wenlock River and flown 77 km north to the Jackson River on the same day (Fig. 1A, Fig. 2A). This crocodile remained in the Jackson River system and around the river mouth for 13 days before travelling 99 km in 15 days, via the coastline, to return to his site of capture on 20 September 2004. During its travel back to the capture site, consecutive positional fixes showed him travelling at least 16.5 km in a single day (Fig. 2B). Once back in the Wenlock River, he moved about within this river system for the remainder of the tracking period, often travelling more than 3 km in a day (Fig 2B).

Crocodile B was captured on 14 September 2003 in the Nesbit River, transported 52 km south and released into the ocean later on the same day (Fig. 1A & Fig. 2C). This crocodile remained in the vicinity of the release site for just over 2 weeks before making a direct journey over 5 days along the coastline back to the Nesbit River (Fig. 2C). Consecutive positional fixes showed that he covered distances in excess of 8 km in a day. Once back in the Nesbit River, Crocodile B remained there for as long as signals continued to be received.

Crocodile C was captured on 16 August 2004 in the Wenlock River and flown overland, 126 km due east to Temple Bay where he was released into the ocean later in the same day (Fig. 1A, Fig. 3A). For more than three months, this crocodile remained in the broad vicinity of his release location, except for a short excursion southwards. From 3 December 2004 he moved northwards, covering large distances each day (Fig. 3A & B). He rounded the tip of Cape York Peninsula on 14 December and then travelled rapidly south to the site of his capture in the Wenlock River (Fig. 3A), arriving 24 December. The total journey was along 411 km of coastline in 20 days and consecutive positional fixes showed that he often travelled more than 15 km per day and on one day travelled 30.4 km. After returning he remained in the Wenlock River for the remainder of the tracking period, the last signal being received on 22 Jan 2005.

## Discussion

Satellite tracking proved very effective for studying the movements of these large crocodiles, enabling them to be monitored essentially continuously across a wide geographic range and for several months. The animals all moved extensively within river systems, undertook substantial coastal voyages, and displayed remarkable navigational abilities.

All of the crocodiles returned to their sites of capture, even Crocodile C which was translocated a long distance and across a major geographic feature. These observations add to previous data showing the ability and, apparently, an inclination by translocated crocodilians to return ‘home’. Walsh and Whitehead (1993) found that 50% of 48 problem *C. porosus* translocated distances between 20 and 100 km from Nhulunbuy harbour in the Northern Territory, Australia, returned to their original capture locations. The fate of the remainder is unknown. Kay (2004) reported that a juvenile male *C. porosus*, translocated 118 km from Port Wyndham to the Ord River, also in northern Australia, returned after 12 days. On the other hand, hatchling and juvenile *C. porosus* translocated in the Liverpool River system in northern Australia returned equivocal results [5]. Webb *et al*. (1983) translocated 17 adult and sub-adult *C. johnstoni* within the McKinlay River (northern Australia) for 39 river km (30 km direct) and a year later recaptured eight of them, all but one at the original capture site even though there was suitable habitat between the capture and release sites. Homing tendency has also been reported over much shorter distances in *Alligator mississipiensis* [8], [17] and *Caiman crocodilus* [4].

What is different about the present study is that, for the first time in any adult crocodilian, we can report detail of the track taken and the time profile of the homeward journey. The results pose interesting questions. It is noteworthy that all three individuals spent some time at the release point before embarking on an apparently purposeful and direct travel homewards. Could the animals be making an appreciation of local cues and the direction of travel required? Also, all returned to the same place at which they were captured, and none travelled any distance in an inappropriate direction, except for a brief excursion southwards by Crocodile C, the most disrupted individual. Was this movement associated with an assessment of the correct direction home? Our observations clearly imply that crocodilians are skilful at interpreting a suite of complex cues for orientation and navigation, and this aspect of their behaviour demands additional investigation.

This study confirms that the practice of translocating ‘problem’ *C.porosus* to a remote site is very likely to be ineffective. If a problem crocodile animal is living in an area where conflict with humans is likely, then other options need to be employed. Of particular interest were the large distances travelled in a comparatively short time. On their voyages back to their capture sites Crocodile A had an average speed of nearly 7 km day^−1^, Crocodile B more than 11 km day^−1^ and Crocodile C accomplished an average speed of more than 20 km day^−1^ over a period of 20 days (Figures 2, 3). No comparable journeys have been reported previously for any crocodilian. The prevailing view is that substantial energy demands in crocodilians are met anaerobically [18] but in making these journeys, *C. porosus* revealed a high capacity for sustaining prolonged exercise. Combined with the lingual salt glands [19], this attribute imbues this crocodilian with a great capacity for dispersal over vast distances, easily explaining its extensive geographic distribution from India to the Solomon Islands.
